# Supplementary material for: Structure-Guided Identification of Critical Residues in the Vacuolar Cation/Proton Antiporter NHX1 from Arabidopsis thaliana
Source: Plants (Basel). 2023 Jul 26;12(15):2778. doi: 10.3390/plants12152778 (PMC10421325; doi:10.3390/plants12152778)
Supplement: Supplementary file 1 [file plants-12-02778-s001.zip › plants-2478633-supplementary.pdf]

plants

Article

Structure-Guided Identification of Critical Residues in the Vacuolar Cation/Proton Antiporter NHX1 from *Arabidopsis thaliana*

Belén Rombolá-Caldentey[1], Imelda Mendoza[1], Francisco J. Quintero[1] and José M. Pardo[1*]

SUPPLEMENTAL TABLES

Supplemental Table S1. SwissModel best-fitting 3D structures for AtNHX1. Top matches with known Na^+/H^+ exchanger structures (templates) are shown with their PDB code. GMQE (Global Model Quality Estimation) is a quality estimation reflecting the expected accuracy of a model built with that alignment of the template and the coverage of the target. Sequence Identity refers to the percentage of amino acid identity between the query and the template. Sequence Similarity was calculated from a normalized BLOSUM62 substitution matrix. Coverage is the proportion of the full-length target protein that is aligned to the template.

Template	Protein	GMQE	Method	Resolution	Seq Identity	Seq Similarity	Coverage
4cza.1.A/B	PaNhaP	0.45	X-ray	3.20Å	22.11	0.30	0.71
4cz8.1.A/B	PaNhaP,	0.45	X-ray	3.15Å	21.99	0.30	0.71
4cz9.1.A/B	PaNhaP (pH 4)	0.45	X-ray	3.50Å	21.99	0.30	0.71
4czb.2.B/A	MjNhaP1 (pH 8)	0.44	X-ray	3.50Å	18.49	0.28	0.71
4d0a.1.A	MjNhaP1	0.44	2DX	6.00Å	18.49	0.28	0.71
4czb.1.A/B	MjNhaP1	0.44	X-ray	3.50Å	18.49	0.28	0.71
4bwz.1.A	TtNapA	0.40	X-ray	2.98Å	14.68	0.28	0.67
5bz3.1.A	TtNapA (outward - facing)	0.40	X-ray	2.30Å	14.64	0.28	0.67
5bz2.1.A	TtNapA (inward - facing)	0.40	X-ray	3.70Å	15.34	0.28	0.68
4au5.1.A	EcNhaA (low pH)	0.31	X-ray	3.70Å	14.84	0.27	0.58

Supplemental Table S2. Amino acid residues and primers used for generating NHX1 mutant proteins. Bold triplets indicate the location of the modified codon, and the small letter the change introduced.

Final vector	Original residue	Mutated residue	Primer
**pDR-D157N**	Asp157<br>GAT	Asn157<br>AAT	157mut:<br>5′TTGCTGCAACA**aAT**TCAGTGTGTAC<br>157rev<br>5′ATATGGCACCAATAGCAAGATAATC
**pDR-R353L**	Arg353<br>AGA	Leu353<br>CTA	353mut:<br>5′CATGGTTGGA**ctA**GCAGCGTTCGTC<br>353rev:<br>5′ACCAGACCCATTAGGATTGAGC
**pDR-N184D**	Asn184<br>AAT	Asp184<br>GAT	184mut:<br>5′GGGTGTTGTG**gAT**GATGCAAC<br>184rev:<br>5′ TCTCCGAATACAAGACTG
**pDR-D185N**	Asp185<br>GAT	Asn185<br>AAT	185mut:<br>5′GTGTTGTGAAT**aAT**GCAACGTCAGTTG<br>185 rev:<br>5′CTCTCCGAATACAAGACTGTAAAGC
**pDR-R353K**	Arg353<br>AGA	Lys353<br>AAA	353Kfw:<br>CATGGTTGGA**AaA**GCAGCGTTCGTC<br>353Krev:<br>GACGAACGCTGC**TtT**TCCAACCATG
**pDR-R390K**	Arg390<br>AGA	Lys390<br>AAA	390KFw:<br>CTGGTCTCATG**AaA**GGTGCTGTATC<br>390KRv:<br>GATACAGCACC**TtT**CATGAGACCAG
**pDR-N184D-R>K**	Asn184<br>AAT	Asp184<br>GAT	184mut:<br>5′GGGTGTTGTG**gAT**GATGCAAC<br>184rev:<br>5′ TCTCCGAATACAAGACTG

SUPPLEMENTAL FIGURES

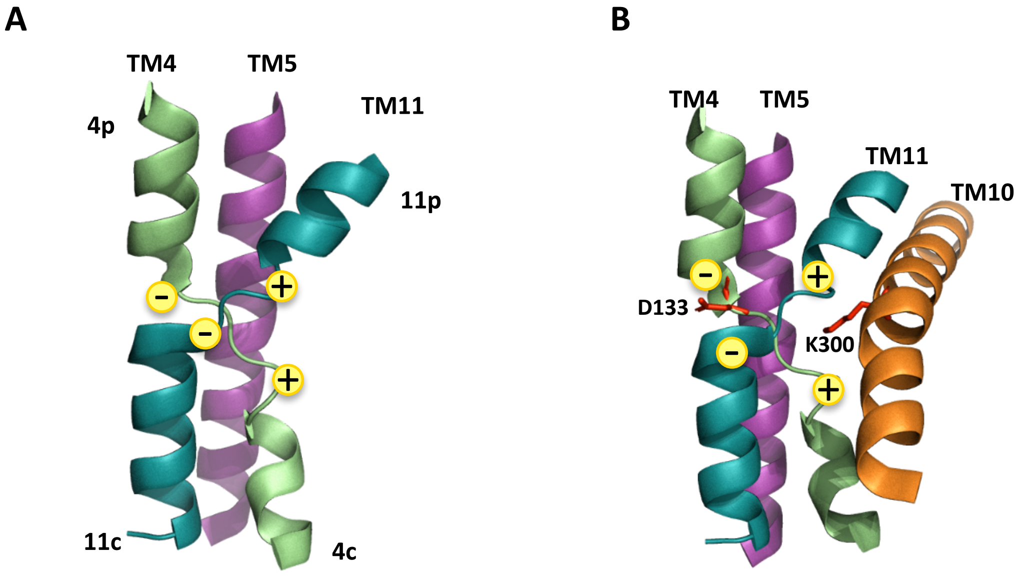

Supplementary Figure S1. Schematic diagram of the NhaA-fold structure. **(A)** TM4 (green) and TM11 (blue) are discontinuous and crossed over the center of the protein, near TM5. The discontinuous helices generate dipoles of opposite charge. **(B)** Asp133 in TM4 and Lys300 in TM10 neutralize the positively and negatively charged helices. Structure is based in the crystal structure of EcNhaA (PDB code 4AU5).

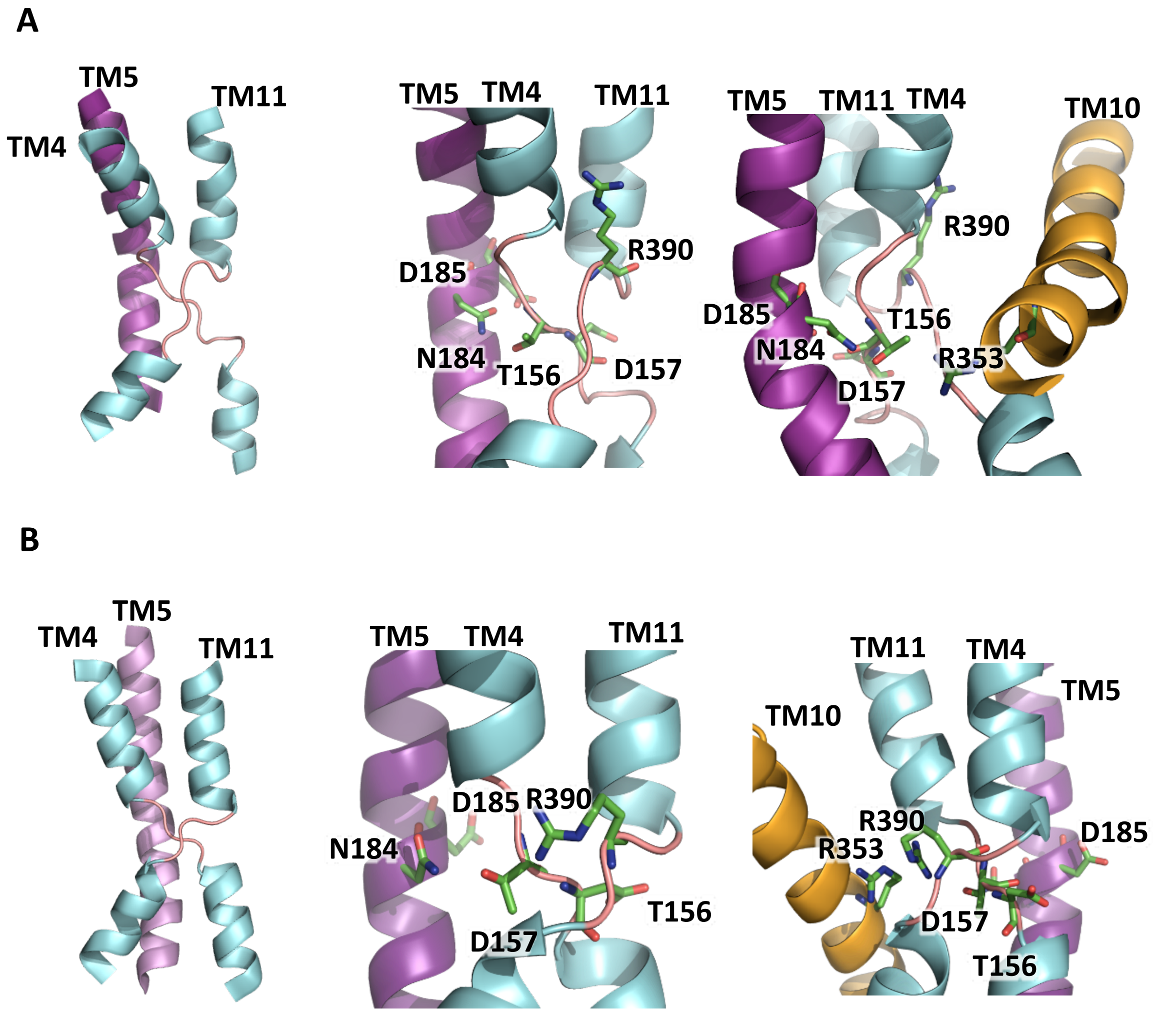

Supplementary Figure S2. Analysis of the NhaA-fold in different models of AtNHX1. Representation of NhaA-fold in the active center of the models of AtNHX1 and close-up view of the cross-over of the extended chains of the highly conserved residues T156 and D157 in TM4, N184 and D185 in TM5, and R390 in TM11. Models were generated using **(A)** MjNhaP1 (4czb) and **(B)** TtNapA (4czb) as templates.

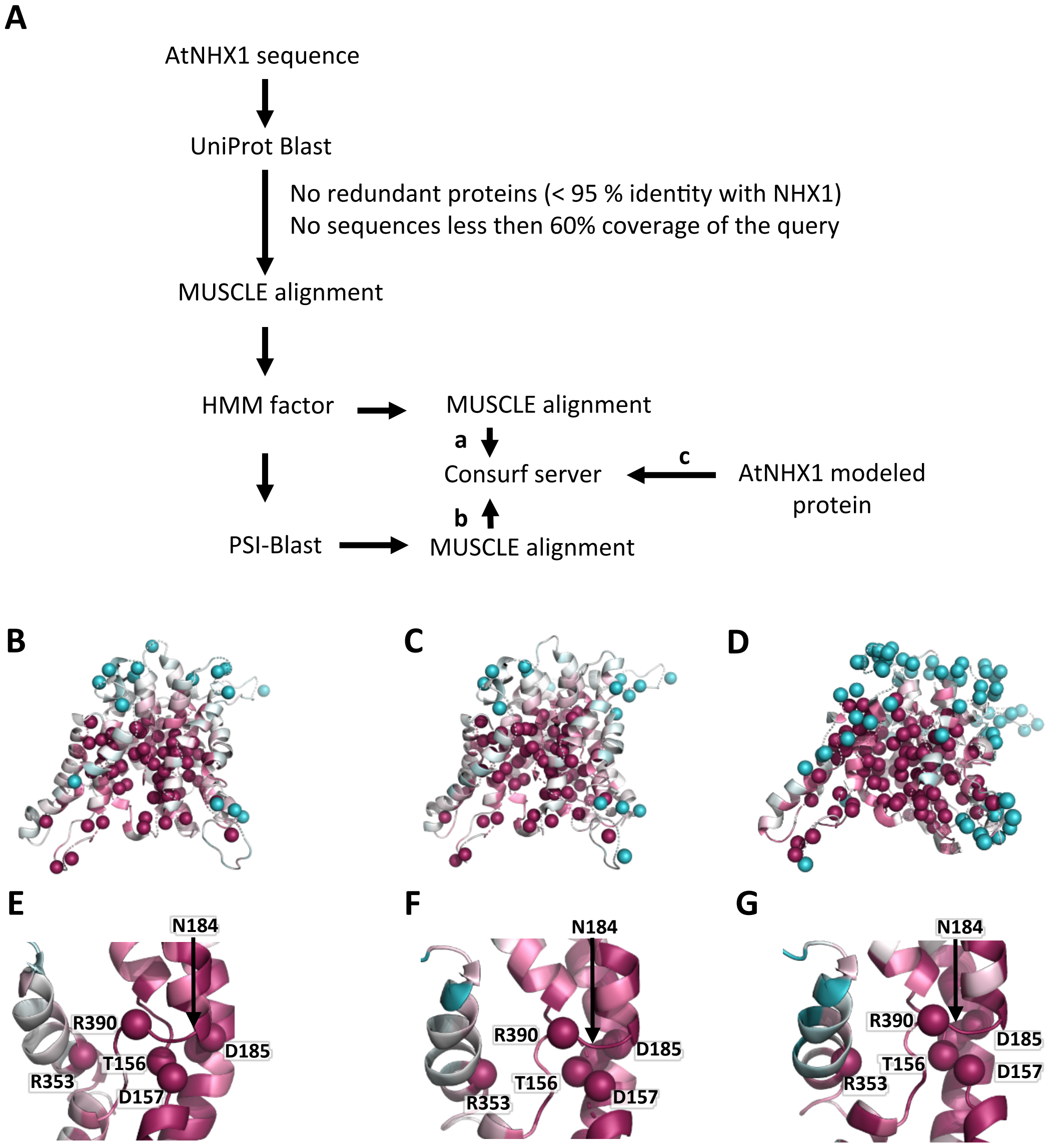

Supplemental Figure S3. **Evolutionary conservation of AtNHX1 residues**. **(A)** Flow chart of the methods followed to obtain the evolutionary conservation profile of AtNHX1 calculated via the ConSurf web-server (http://consurf.tau.ac.il). **(B-G)**. The evolutionary conservation profile of AtNHX1 obtained by the previous methods; **(B)** and **(E)** corresponds to the process following arrow *a*; **(C)** and **(F)** with arrow *b*, and **(D)** and **(G)** with arrow *c*. The profiles are colored according to their conservation-grades using the color-coding bar, indicating turquoise the most variable residues and purple the most conserved. variable-through-conserved. The most variable and most conserved positions of each transporter are shown as spheres. In all three cases the pattern was repeated: a highly conserved intramembranous core while intervening loops and lipid-facing residues are variable. **(H-J)** Representation of the sequences in TM4, 5, 10 and 11 for each process following arrows routes *a*, *b* and *c* respectively. Note the high conservation of the residues in TMs contributing to the NhaA-fold in the active center. TM10 is not highly conserved, except for the R353 residue. The AtNHX1 model shown is based on PaNhaP template.

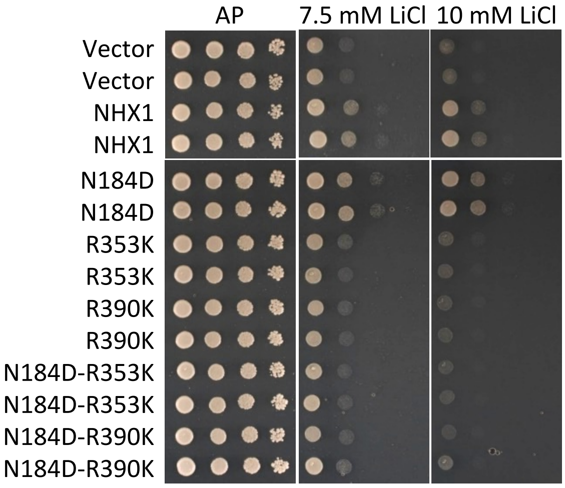

Supplementary Figure S4. Functional assay of AtNHX1 mutant alleles in amino acids at the ion coordination pocket. The cDNAs of wild-type AtNHX1 and the indicated mutant alleles of the indicatd residues were subcloned into the yeast expression vector pDR195 and transformed into the AXT3K (*Δena1-4 Δnha1 Δnhx1*). Overnight cultures were normalized in water to OD_{600} of 0.5. Aliquots (5µL) from normalized cultures and 10-fold serial dilutions were spotted onto AP medium plates. Plates were incubated 2-3 days at 30ºC and pictured.

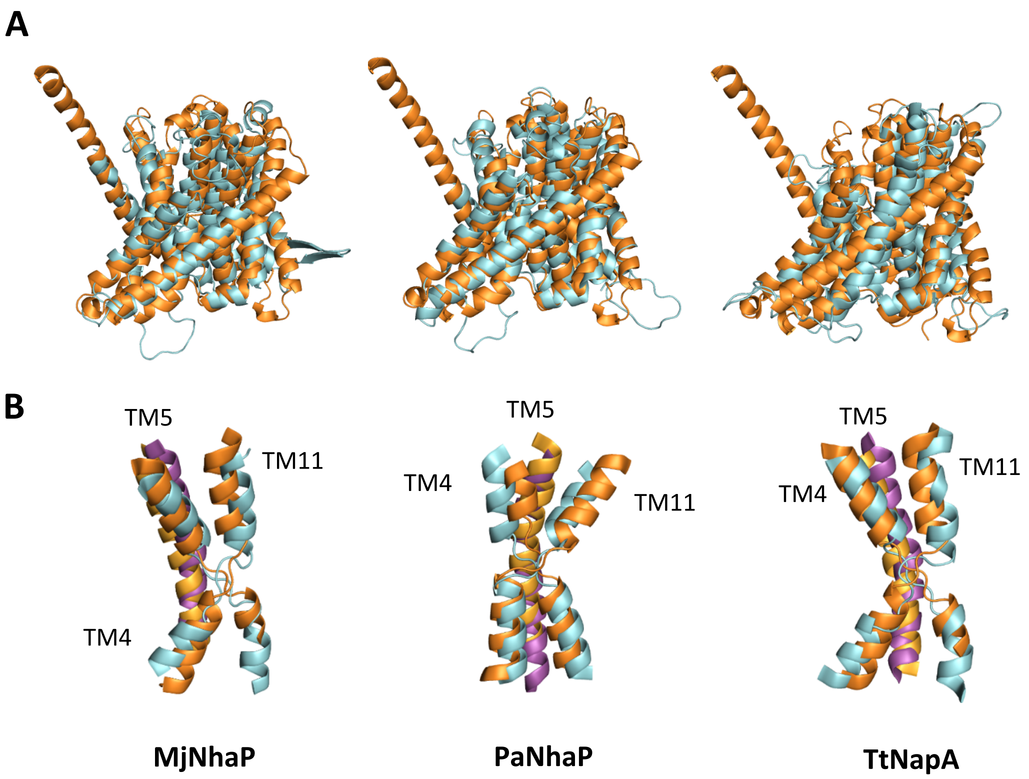

Supplementary Figure S5. Overlay of the AtNHX1 three-dimensional structures. Structure generated by the AlphaFold software (orange) merged with the different models generated using the proteins in the Swiss-model repository as templates (blue). **(A)** Overlay of the pore domains. **(B)** Overlay of the Nha-fold structures. The names at the bottom indicate template used in each case.

A

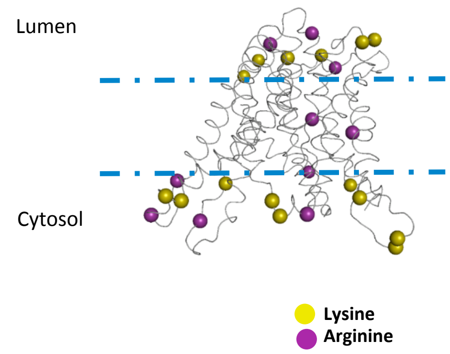

Model	Total K+R	Inside	Outside	Intramolecular
**TtNapA**	27	15	8	3
**MjNhaP1**	27	15	9	2
**PaNhaP**	27	15	9	2
**EcNhaA**	20	2	14	4

B

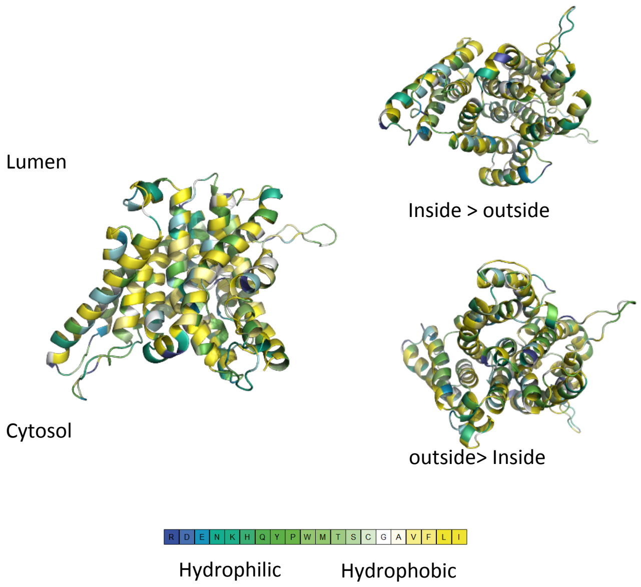

Supplementary Figure S6. **Validation of the modeled AtNHX1 structure.** The AtNHX1 model represented corresponds to the PaNhaP template**. (A)** The Cα of lysines (yellow) and arginines (purple) are highlighted in the structure to determine their distribution in the protein**.** Except for the model based on EcNhaA, other modeled structures of AtNHX1 are in accordance with the ‘positive-inside’ rule. **(B)** Distribution of the amino acids according to their hydrophobicity across the AtNHX1 structure based on the PaNhaP template. Amino acids are colored based on the hydrophobicity scale of Kessel and Ben-Tal.
